# Research Activity and the New Pedagogy: Why Carrying Out Research Is Essential for Effective Learning

**DOI:** 10.3389/fpsyg.2017.01838

**Published:** 2017-10-19

**Authors:** Patrick Tissington, Carl Senior

**Affiliations:** ^1^Department of Organizational Psychology, Birkbeck, University of London, London, United Kingdom; ^2^Department of Psychology, School of Life and Health Sciences, Aston University, Birmingham, United Kingdom; ^3^University of Gibraltar, Gibraltar

**Keywords:** teamworking, transferable skills, the Idea of a University, pedagogy, research training

The modern-day university is a thoroughly complex affair that comprises of numerous interlocking research activities that inform the delivery of an equally complex portfolio of learning programs (Kerr, 1963; Krücken et al., 2007). This contemporary model of a university is a far cry from university education envisioned by the noted educational philosopher Cardinal John Henry Newman^1^. In his seminal paper on the nature and purposes of a University, Newman was clear that a university should be a place where students would acquire a liberal education that would enable them to graduate and to “… see things as they are, to go right to the point, to disentangle a skein of thought to detect what is sophistical and to discard what is irrelevant.” (Newman and Svaglic, 1982, p. 6). Although, Newman's philosophy is at the heart of universities across the globe, the day-to-day reality of delivering his core principles within the context of a modern-day university is such that a casual observer might not see how a graduate should be able to develop the skills that Newman originally espoused. However, here we argue that by engaging students at the very heart of the research activity that is regularly carried out in a contemporary university it is indeed possible for Newman's original vision to be realized.

That said, it is worth considering Newman's philosophy in the context of the period when there were very few universities, mostly of ancient origins, and were dedicated to the education of elite “gentlemen.” The curriculum was a loosely structured experience of academic teaching that centered on political debate, religious knowledge, and physical pursuits (de Ridder-Symoens, 1996). From the 1850s influenced by Newman and others, in the UK a small number of civic universities was created following the examples in the UK of Durham, Manchester, and London where students were prepared for their role in the world with science, engineering, and politics appearing on the curriculum. The relevance to the world of work was more clearly aligned with Newman's original ideals with preparation for employment being delivered via critical thinking rather than professional knowledge.

Following the Second World War, universities widened their recruitment pool and grew as a more egalitarian world was sought. There was a wider remit and a sense of state funded paternalism where students were the grateful recipients of whatever learning experience the university's academics considered appropriate.Later there was a movement toward collectivist ideals of the 1960s where universities were seen by activists and some academics as being democratic communities of learning where students and staff had an equal role. In some ways, these ideas were the basis of widening participation in the 80s and 90s culminating for example in the UK with ambitious aims for university attendance of 50% (Smithers, 2001). It was at this time when the emergence of the specialist teaching university started to emerge where the onus was on the completion of effective learning by students and not so much on the creation of knowledge through research.

The gradual evolution of the global HE sector into a two-part system can now be seen with the emergence of groups such as the Ivy League system in the US, the Russell Group in the UK and the Group of Eight in Australia. These groups consist of universities that claim to be leading in research excellence in a particular area (see e.g., Williams et al., 2007). Reputationally it makes perfect sense to be considered as a research active university than a teaching active university (Wuchty et al., 2007). Most of the professoriate consider their professional identity to be more aligned toward their research activity than to teaching (Harris, 2005). There is also a greater opportunity to secure more institutional funding. Indeed, financial support in the form of private endowments for institutes such as Harvard and Yale Universities in the US are substantial^2^.

That said, even these research intensive universities are sensitive to the vagaries of market forces that would shape the delivery of their core product—i.e., excellence in a researchinformed learning experience. Thus in light of ever-growing market complexity it remains to be seen whether or not the provision of research informed teaching and indeed research as an activity is still the *raison de etre* in the modern university. It may come as a surprise to many that the inclusion of research activities within the portfolio of a university was not the main driver for their creation. Newman was clear in his disdain for research in his early writings and initially saw research activity as being completely distinct from an effective university education. Indeed, he was clear in the role that research activity had in the development of a University e.g., “*Intellectual training was the primary duty of a university. Research is not training, but rather it is philosophical or scientific discovery or “advancement”… if its object were scientific and philosophical discovery, I do not see why a University should have students*” (Newman and Svaglic, 1982, p. 1).

The separation of research and teaching activities is clearly not in the market interests of a modern-day university. Here we argue that a university should not only facilitate the various research activities of the professoriate, but that the role of the student should be placed firmly at the center of such activities.

Notwithstanding Newman's early concerns on the separation of research and teaching, there is a significant benefit to be had with the research *activity* itself (Hathaway et al., 2002). Scholars who are engaged in the activity of scientific discovery are in general at the forefront of scientific thinking to ensure that they can address a specific research question (Jones and Moreland, 2003). These individuals tend to be flexible minded and open to feedback and by its very nature they are used to the experience of failure which quite paradoxically drives innovation and an enterprising mind-set (Cope, 2011). Clearly, the modern-day undergraduate would have much to benefit be spending time with such individuals. Yet this is not a one-sided relationship with the students developing a unique transferable skill set by being embedded within a research culture. The researchers themselves would benefit from the exposure to the constant inquiry that arose by carrying out their activities alongside students which would ingrain a collaborative research culture into the notion of the scholarly community (Shulman, 1993).

In our earlier work we have also found that students expected to be part of the research culture of the university and report the experience of working side-by-side with a member of the professoriate as one of key experiences of a university education (Towl and Senior, 2010). Here, they regarded research activity as being a fundamental aspect of the university experience. Moreover, the expectation to be trained in contemporary research techniques and the development of a sense of community development was the key extrinsic motivator for participation. The importance of taking part in research activity was first highlighted by in the 1998 report commissioned by the Carnegie Foundation for the Advancement of Teaching in the United States of America. For universities to deliver a truly authentic learning universities would need “*…to be able to give their students a dimension of experience and capability they cannot get in any other setting*…” (Boyer, 1996, p. 27). Boyer showed that learning would be best facilitated by a culture based on discovery that was guided by mentoring rather than solely on the traditional didactic transmission of information. Unfortunately the presence of such research based partnerships between the professoriate and student is not the current orthodoxy—a situation that led to noted Nobel Laurates decrying the separation of active research experience from the student cohort (Hubel, 2009). Placing research activity at the very heart of student culture could be a relatively straightforward way to ensure that the modern day undergraduate student benefits from focused mentoring.

In considering the above, there is clearly a need for institutional managers to facilitate research activity as well as encourage students to participate fully with such activities. However, there is a secondary benefit that students can acquire via participation in research activity that is now discussed. This will inform a complete understanding of the role that research activity plays as an effective learning process within higher education and further place Newman's core ideals of enabling students to detect sophistry in any argument firmly at the center of all contemporary university activity.

Research activity requires a unique set of professional skills that ultimately benefit the student in the post-graduation workplace. These transferable skills, such as project management and team skills, are vital for effective employment and make an excellent contribution to the professional skillset that undergraduate students expect to develop within HE (Senior et al., 2014). And yet there is only sporadic effort at best to ensure that all students have the opportunity to experience research activity.

Effective research activity is rarely carried out in isolation so much so that it has now become the norm for the best quality research to be carried out in teams (Tissington and Senior, 2013). The tacit skill set that is developed is something that is eminently transferable into the world of work. However, it is rare for students to be provided with a framework to operate to when working in groups and standard pedagogic practice to develop team skills such as group assignments are seen as learning by doing and not reflective. Participation in research activity is one way in which the development of reflective team skills can be is embedded within the curriculum^3^.

These “Non-Technical Skills” are regarded as being crucial for professional teams across professions and in extreme environments such as aviation and operating theaters is regarded as crucial (Salas et al., 2013). However as they might be referred to in universities as being “non-academic skills” there is a risk that they are perceived as being of less value by the students. However, by incorporating research activity into the curriculum students will benefit from by developing both technical and non-technical skills. The advantage of such an approach is that the development of team skills is broadly similar regardless of the activity that the student undergoes and that the students are not aware that developing this important skillset (Senior and Howard, 2014). The critical element to ensure effective learning is that students are actively encouraged to participate in research activity throughout the course of their learning.

Research activity provides a valid opportunity for the learning of team skills and by providing learning about the evidence base for teams (e.g., West, 2012), students will discover ways of working to avoid pitfalls of teamworking frequently experienced in the workplace. Our recommendation is for students to have development sessions to foster team skills before and during these research projects. But we specify that this training would be based on firm evidence so (inter alia) students could learn classic findings such as groupthink (Janis, 1971) as well as recent evidence about conflict (De Dreu and Weingart, 2003), the pre-requisites for “real teams” (Lyubovnikova et al., 2015) and how to avoid social loafing (van Dick et al., 2009). In this way, students would see the value of the application of research to their practice as well as learning concepts of teamworking which would then be applied in team based research projects.

These are important transferable skills that students expect to acquire with a university education. However, this is not the sole benefit for engaging with research activity. As is described above those students who engage with research activity also experience a greater degree of affiliation with their professoriate and engagement with their studies (Towl and Senior, 2010). These are the core skills that will ultimately ensure that the student will be able to detect sophistry and focus on what is relevant to ensuring success at university and in their careers—whatever these may be.

## Author contributions

All authors listed have made a substantial, direct and intellectual contribution to the work, and approved it for publication.

### Conflict of interest statement

The authors declare that the research was conducted in the absence of any commercial or financial relationships that could be construed as a potential conflict of interest.

## Footnotes

^1^See John Henry Newman's seminal essay “*The Idea of a University” (1852)* for his liberal ideal of a university.

^2^Harvard University has an endowment fund which by 2016 was worth $35 Billion: http://www.hmc.harvard.edu/docs/Final_Annual_Report_2016.pdf which places it ahead in wealth of countries such as Gibraltar ($1.8 Billion) the Seychelles (2.5 Billion) and even Nicaragua (33.5 Billion) Source: The CIA Factbook: https://www.cia.gov/library/publications/the-world-factbook/.

^3^The utility of team reflection is something that has long been realized and emerges in a variety of different and quite unique settings (e.g., Leeson, 2007) see Knight and Senior (2017) for a more detailed description of this concept in a contemporary organizational setting.
